# Stability of Metabolomic Content during Sample Preparation: Blood and Brain Tissues

**DOI:** 10.3390/metabo12090811

**Published:** 2022-08-29

**Authors:** Maxim V. Fomenko, Lyudmila V. Yanshole, Yuri P. Tsentalovich

**Affiliations:** 1 Laboratory of Proteomics and Metabolomics, International Tomography Center SB RAS, Institutskaya 3a, 630090 Novosibirsk, Russia; 2 Department of Physics, Novosibirsk State University, Pirogova 2, 630090 Novosibirsk, Russia

**Keywords:** sample preparation, human blood, rat brain, metabolomics, NMR spectroscopy

## Abstract

Thermal and enzymatic reactions can significantly change the tissue metabolomic content during the sample preparation. In this work, we evaluated the stability of metabolites in human whole blood, serum, and rat brain, as well as in metabolomic extracts from these tissues. We measured the concentrations of 63 metabolites in brain and 52 metabolites in blood. We have shown that metabolites in the extracts from biological tissues are stable within 24 h at 4 °C. Serum and whole blood metabolomes are also rather stable, changes in metabolomic content of the whole blood homogenate become apparent only after 1–2 h of incubation at 4 °C, and become strong after 24 h. The most significant changes correspond to energy metabolites: the concentrations of ATP and ADP decrease fivefold, and the concentrations of NAD, NADH, and NADPH decrease below the detectable level. A statistically significant increase was observed for AMP, IMP, hypoxanthine, and nicotinamide. The brain tissue is much more metabolically active than human blood, and significant metabolomic changes occur already within the first several minutes during the brain harvest and sample homogenization. At a longer timescale (hours), noticeable changes were observed for all classes of compounds, including amino acids, organic acids, alcohols, amines, sugars, nitrogenous bases, nucleotides, and nucleosides.

## 1. Introduction

Metabolomics is the youngest discipline among other “-omics”—genomics, transcriptomics, proteomics, and during the last decade it has developed very rapidly. Nowadays, metabolomics becomes one of the most called-for techniques for studying biochemical processes in a large variety of biological subjects, including cell cultures and tissues from plants, animals, and humans. Since the metabolomic profile of a tissue reflects the metabolism of this tissue and of the whole body, metabolomics plays an especially important role in understanding the molecular mechanisms of diseases, and in the development of novel methods for diagnosis and treatment of a wide range of human diseases. Usually, the metabolomic analysis is performed with the use of two analytical platforms: nuclear magnetic resonance (NMR) spectroscopy and liquid or gas chromatography followed by mass-spectrometric analysis (LC-MS or GC-MS). The major advantage of the LC-MS method is a high sensitivity, which allows for the detection of hundreds of metabolites in a single run. The disadvantage of the method is that the intensities of the MS signals depend on the ionization efficiency of every particular metabolite, which makes the quantitative measurements of metabolite concentrations in a sample rather difficult and not always reliable [1,2]. For this reason, the LC-MS method is often used for semi-quantitative measurements, i.e., the comparison of metabolomic profiles of experimental and control samples. NMR-based metabolomics [3] is less sensitive: typically, one can detect 50-80 metabolites in a biological sample. However, the intensities of all NMR signals are proportional to the compound concentrations, and quantitative determination of the metabolite concentrations is straightforward and simple.

Regardless of the analytical platform used, the most crucial and important part of the metabolomic analysis is the sample preparation. It has been estimated that in clinical practice, the pre-analytical phase accounts for up to 80% of all testing errors [4]. These errors originate from three major sources: incomplete metabolite extraction, chemical degradation of metabolites during the sample preparation, and metabolite transformation due to enzymatic reactions. The pre-analytical phase includes tissue homogenization and metabolite extraction, and the efficiency of different homogenization and extraction methods has been evaluated in a number of research papers and reviews [5,6,7,8,9,10,11,12,13,14,15]. Chemical and biochemical metabolite transformation can be minimized by following two simple rules: sample homogenization and extraction should be performed at low temperatures immediately after the sample collection or thawing of the frozen samples, and the enzymatic activity should be quenched completely as soon as possible [5,6,7]. Unfortunately, compliance with these rules is not always applicable. For example, tissues or tissue homogenates can be used for studying the effect of external factors (chemical agents, UV irradiation, etc.) on biochemical processes [16,17,18,19] in a tissue. Strict compliance with the protocols of sample collection and treatment might be difficult in field studies. The analysis of serum samples often requires a 30–60 min delay for sample clotting. In all these cases, some delays in enzymatic activity quenching and metabolite extraction are unavoidable, and it is important to know which metabolites undergo transformations, and the rate of these reactions. It should be noted that the metabolomic composition and the enzymatic activity of different tissues differ, and the protocols developed for one type of tissue might be not quite valid for other types.

In many metabolomic studies involving the analysis of intracellular components, the important issue is also the method of tissue homogenization and cell lysis [20]. The most popular approaches are either application of freeze-thaw cycles or lysis by chemical agents such as cold methanol or acetonitrile combined with mechanical homogenization. The advantage of the second approach is that high concentrations of cold methanol not only cause the disruption of the cell membranes due to osmotic stress and temperature shock but also quench the enzymatic activity of both extra- and intracellular proteins. The freeze-thaw method implies that during the thaw period, the enzymatic activity remains unquenched, which may result in undesirable metabolomic changes.

In the present work, we studied the metabolite stability in extracts and homogenates from two types of tissues—human blood and rat brain. One can expect that the metabolic activity in these types of tissues differs significantly. The human blood is a fluid most frequently used for metabolomic studies, clinical studies in particular. From the viewpoint of metabolic activity, this tissue (especially blood serum) is considered relatively inert [21,22]. Opposite to blood, the brain features very high metabolic activity and a high rate of post-mortem metabolomic transformations [23]. In this study, we incubated the tissue extracts and homogenates at 4 °C, and the changes in the metabolomic composition of the samples were detected by the NMR method. We also compared different methods of whole blood cell lysis. The major goals of the study are to estimate the metabolome stability in serum, whole blood, and brain, and to evaluate the rate of the metabolomic changes in tissue homogenates and extracts.

## 2. Materials and Methods

### 2.1. Chemicals

Solvents methanol and chloroform (HPLC grade) were from Panreac (Barcelona, Spain), and deuterated water (99.9%) was from Armar Chemicals (Aargau, Switzerland). We used Ultra Clear UV plus TM water system (SG water, Munich, Germany) to deionize H_2_O to 18.2 MΩ.

### 2.2. Sample Collection

All procedures related to human blood study were performed according to the Declaration of Helsinki—ethical principles for medical research involving human subjects. Blood samples were obtained from two healthy donors with written informed consent. We used blood from the first donor for metabolomic analysis of serum, and from the second—for whole blood studies. Blood samples were taken from antecubital vein. Blood samples obtained from the first patient were immediately centrifuged (3000 g, 10 min), the obtained plasma was frozen and stored at −70 °C until analyzed. The samples were thawed immediately before analysis or incubation (see below), and the fibrin clot was removed. Whole blood samples from the second donor were used immediately after the collection to obtain blood extracts and homogenates.

We obtained 3-month-old Wistar rats from the Institute of Cytology and Genetics SB RAS. All procedures were carried out in accordance with Directive 2010/63/EU of the European Parliament and the Council of the European Union of 22 September 2010 on the protection of animals used for scientific purposes. Rats were decapitated, the rat sculls were open, the brain was removed, weighed, frozen in liquid nitrogen, and stored at −70 °C until analyzed. The time interval between the animal death and brain freezing was approximately 3 min.

### 2.3. Extract and Homogenate Preparation

Blood extracts were obtained by addition of 600 µL of cold (−20 °C) methanol into vial with 300 µL of freshly obtained blood. We homogenized the mixture with a TissueRuptor II homogenizer (Qiagen, Venlo, Netherlands), and then added 300 µL of cold H_2_O and 600 µL of cold chloroform. The sample was placed in a shaker for 10 min at +4 °C, kept at −20 °C for 30 min, and then centrifuged at 16,100 g, 4 °C for 30 min. Upper (MeOH-H_2_O) fraction was collected.

We obtained whole blood homogenates by three freeze-thaw cycles of freshly collected blood samples (300 µL) followed by homogenization.

To obtain brain extract, 11.5 mL of cold (−20 °C) methanol was added to 1 g of frozen brain sample, the mixture was homogenized. Then we added 5.6 mL of H_2_O and 12 mL of cold chloroform, placed the sample in a shaker for 10 min at 4 °C, then placed it at −20 °C for 30 min, and centrifuged at 16,100 g, 4 °C for 30 min. Upper fraction was collected.

Brain homogenate was obtained by thawing the brain tissue (1 g) in 4.6 mL of H_2_O (at room temperature) followed by homogenization with the TissueRuptor II homogenizer.

### 2.4. Sample Incubation and Preparation for NMR Analysis

Serum, extract, and homogenate samples were incubated at 4 °C with the use of Memmert INB 200 Incubator (Schwabach, Germany). After removal from the thermostat, serum samples (150 µL) were extracted by addition of 150 µL of H_2_O, 300 µL of cold methanol, 300 µL of cold chloroform. 600 µL of cold methanol, 600 µL of cold chloroform, and 300 µL of H_2_O were added to 300 µL of the blood homogenate, and 800 µL of cold methanol and 800 µL of cold chloroform were added to 400 µL of the brain homogenate. The samples were placed in the shaker for 10 min at 4 °C, kept at −20 °C for 30 min, and centrifuged at 16,100 g, 4 °C for 30 min. Upper fraction was collected.

All extracts were vacuum dried and stored at −70 °C until metabolomic analysis. For NMR analysis, dry extracts were re-dissolved in 600 µL of 50 mM deuterated phosphate buffer (pH 7.2) containing 2 × 10^−5^ M sodium 3-trimethylsilylpropane-1-sulfonate (DSS) as an internal standard.

### 2.5. NMR Measurements

We performed all ^1^H NMR measurements at the Center of Collective Use “Mass spectrometric investigations” SB RAS with the use of a NMR spectrometer AVANCE III HD 700 MHz (Bruker BioSpin, Ettlingen, Germany) as described in [24]. Each spectrum was obtained with 64 accumulations and a repetition time of 20 s between scans. The obtained spectra of blood and brain extracts are similar to the spectra published by our [25,26] and other [27,28,29,30] research groups. The signal identification in NMR spectra was based on our previous studies [26,31,32] as well as on the spectral data from other laboratories [29,30,33]. The concentrations of metabolites in NMR samples were determined by the peak area integration, respectively, to the internal standard DSS, and then recalculated into metabolite concentrations in a tissue (in nmoles per gram of the sample wet weight).

### 2.6. Statistical Analysis

Statistical treatment of quantitative metabolomics data—calculation of the Mann–Whitney U test—was performed on the MetaboAnalyst 5.0 web platform (www.metaboanalyst.ca (accessed on 1 July 2022) [34]).

## 3. Results

### 3.1. Incubation of Serum at 4 °C

We placed seven vials, each containing 0.15 mL of freshly thawed serum, into the thermostat at 4 °C. The samples were removed from the thermostat after the time intervals of 0, 0.5, 1, 2, 3, 5, and 24 h, and then they were immediately subjected to the H_2_O/MeOH/CHCl_3_ extraction. NMR spectra of the serum extracts were obtained, and 52 metabolites in the spectra were reliably identified and quantified. We found that the metabolite levels in the samples practically did not change during the incubation; mean values of the metabolite concentrations (three replicates) are collected in Table 1.

Practically all metabolites detected in serum are chemically stable: the content of nucleotides, which are usually considered as unstable compounds, was too small for the reliable detection by the NMR method. To check the stability of nucleotides in serum, we added 0.8 mM of oxidized and reduced nicotinamide adenine dinucleotide (NAD and NADH), cytidine monophosphate (CMP), and uridine monophosphate (UMP) into serum, and performed the same incubation experiment monitoring the levels of nucleotides. We found that within the first five hours of incubation at 4 °C, the concentrations of nucleotides in serum did not change. After 24 h of incubation, the concentration of NADH, CMP, and UMP decreased by approximately 20–30%.

### 3.2. Whole Blood Extraction: Cryogenic Lysis versus Methanol Lysis

We placed freshly obtained human blood in two vials (each containing 300 µL of blood) and applied two methods of blood cell lysis. Blood in the first vial was frozen in liquid nitrogen and then thawed in water at room temperature. This procedure was repeated two times, and then, after mechanical homogenization with the addition of 300 µL of deionized water, 600 µL of cold (−20 °C) methanol was added. The total time of freeze-thaw cycles can be estimated as 12–15 min. In the second vial, we added 600 µL of cold methanol immediately without freeze-thaw cycles, after which we also homogenized the tissue. Below, the first sample will be referred to as the “blood homogenate at zero time”, and the second sample—as the “blood extract at zero time”. After obtaining dry extracts and re-dissolving them in the deuterated buffer, the samples were subjected to the NMR-based metabolomic analysis. This experiment was repeated three times, the average metabolomic data (mean ± sd) are presented in Table 1, and the NMR spectrum with the signal assignment is shown in Figure S1. It should be noted that the samples of serum and whole blood were obtained from different donors, so the direct comparison between these types of samples is not relevant.

The comparison of metabolomic compositions of the blood homogenates and extracts at zero time does not reveal significant differences between the two types of sample preparation: the metabolomic profiles of the samples practically coincide.

### 3.3. Incubation of the Whole Blood Extracts at 4 °C

The incubation of the whole blood extracts was performed in the same way as that of serum. We incubated three groups of samples, each containing seven extract samples, at 4 °C for 0, 0.5, 1, 2, 3, 5, and 24 h, and then performed the metabolomic analysis for all 21 samples. It was found that within the first 5 h of incubation, the metabolomic composition of the extract practically did not change. After 24 h of incubation, we observed a statistically significant decrease in GSH, ergothioneine, ADT + ATP, and NADH, and an increase in acetate, pyroglutamate, pyruvate, and AMP.

### 3.4. Incubation of the Whole Blood Homogenates at 4 °C

**Figure 1:**
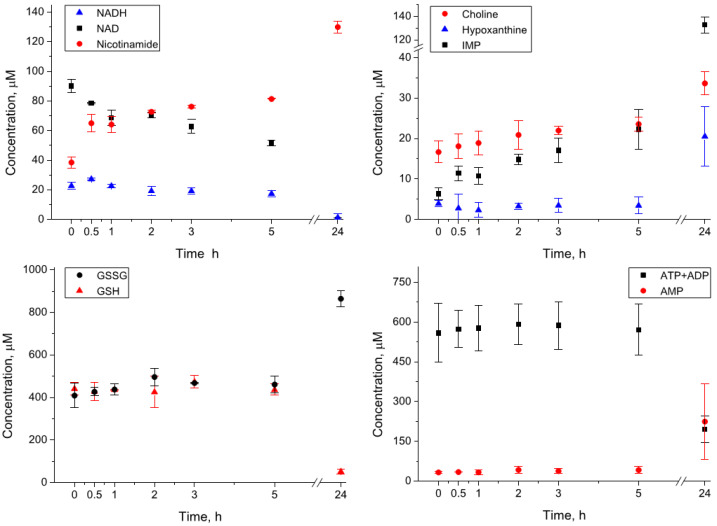
Kinetics of metabolite concentrations in the whole blood homogenates during the incubation at 4 °C.

Whole blood homogenates (three replicas) obtained by the blood cryogenic lysis were incubated at 4 °C for 0, 0.5, 1, 2, 3, 5, and 24 h. The preliminary measurements showed that the level of NADH in the blood is too low for reliable quantification; in order to monitor the stability of this important metabolite, 100 µM NADH were added to homogenates before incubation. The most significant changes were found for compounds corresponding to the “Nitrogenous bases, nucleotides, nucleosides” group (Table 1). A significant portion of NADH decayed already during the sample preparation, and the NADH concentration at zero time was found to be fourfold lower than expected. The NADH decay gave rise to the formation of its decomposition products, NAD, ADP ribose, and nicotinamide: the levels of these compounds in homogenates at zero time was significantly higher than that in extracts. The changes continue at the early stage of the incubation (first five hours) and become strong after 24 h (Figure 1). In particular, the concentrations of ATP and ADP decrease fivefold, and the concentrations of NAD, NADH, and NADPH decrease below the detectable level. At the same time, we observed a significant increase in AMP (sevenfold), IMP (twentyfold), hypoxanthine (fivefold), and nicotinamide (threefold). Among other compounds, we found significant growth for glucose, choline, oxidized glutathione GSSG, lactate, pyroglutamate, pyruvate, and succinate. The decrease was observed for GSH, mannose, and α-ketoisovalerate. We also observed a moderate increase in the levels of many amino acids, but this increase was not always statistically significant (Table 1). The complete set of kinetic data is presented in Table S1.

### 3.5. Brain Extraction: Homogenization Followed by Enzyme Quenching versus Enzyme Quenching Followed by Homogenization

Frozen samples of the rat brain were placed in two vials. The sample in the first vial was thawed by the addition of water at room temperature (4.6 mL per 1 g of tissue), homogenized for 10 min, and then cold methanol was added. In the second vial, methanol was added before homogenization instead of water. After that, the samples were treated in the same way as the blood samples, including the addition of cold chloroform, extraction, centrifugation, drying of the water-soluble fraction, and NMR-based metabolomic analysis. We identified and quantified the total of 63 compounds in the brain samples (Figure S2). The experiment was repeated three times, the obtained data are given in Table 2.

Apparently, during the sample homogenization without the enzymatic activity quenching by methanol, the tissue metabolomic composition underwent significant changes. The most drastic changes we observed for compounds related to the group “Nitrogenous bases, nucleosides, nucleotides”. In particular, IMP, NAD, NADH, NAPH, GMP, and UMP found in the samples with immediate quenching were not detected in the samples with delayed (ten minutes) quenching. We also observed significant decays for adenosine, guanosine, and uridine phosphates ATP, ADP, AMP, and GTP. At the same time, a strong increase in the samples with delayed quenching was found for nitrogenous bases and nucleosides: hypoxanthine, guanosine, xanthine, inosine, adenosine, cytidine, uracil, and uridine. Less impressive, but still statistically significant changes were also found for other groups of metabolites. The concentrations of the majority of amino acids in the samples with delayed quenching are higher than that in the samples with immediate quenching. The only exception is asparagine; its level in the samples with delayed quenching underwent a threefold decrease. We should also note the formation of several organic acids (pyroglutamate, acetate). The levels of compounds from the TCA cycle also undergo noticeable changes: the concentration of fumarate increases, while the level of succinate decreases. A significant increase was observed in the levels of glycerol and sugars glucose and mannose: in the samples with immediate quenching, the concentrations of these metabolites were either low or even below the detectable level.

### 3.6. Incubation of the Brain Extracts at 4 °C

Incubation of the brain extracts was performed in the same way as we did with the serum and whole blood samples: seven vials with 2 mL of the brain extract were incubated at 4 °C, the samples were removed from the incubator after 0, 0.5, 1, 2, 3, 5, and 24 h, and subjected to the NMR-based metabolomic analysis. We found that the levels of almost all metabolites did not change during 24 h of incubation at 4 °C, including potentially unstable compounds such as nucleotides, NADH, and GSH (Table 2). Noticeable decay (approximately twofold) was observed for only one compound, NADPH.

### 3.7. Incubation of the Brain Homogenates at 4 °C

**Figure 2:**
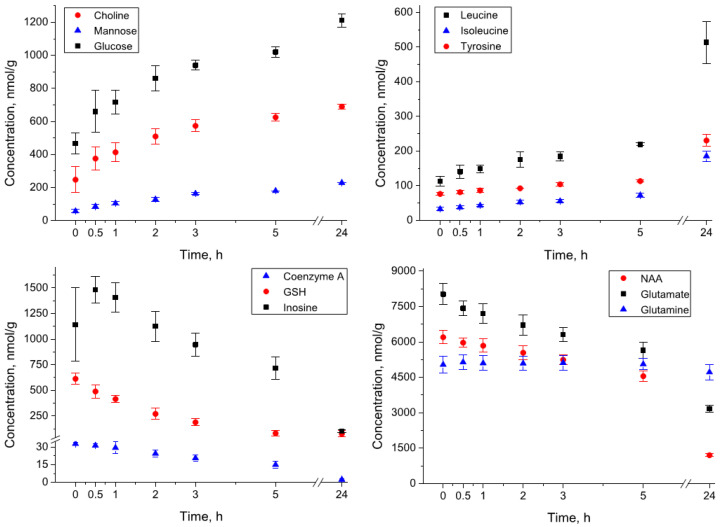
Kinetics of metabolite concentrations in the rat brain homogenates during the incubation at 4 °C.

We performed similar kinetic measurements with the brain homogenates. As we reported above (Section 3.5. Brain extraction: homogenization followed by enzyme quenching versus enzyme quenching followed by homogenization), drastic changes in the metabolomic composition of the brain homogenate occur already during the sample homogenization (approximately ten minutes). The subsequent incubation intensifies these changes (Table 2). In particular:

*Amino acids.* The levels of the majority of amino acids increase during the incubation. The scale of the increase during 24 h of incubation for different amino acids varies from twofold (e.g., alanine, serine, tryptophan) to 5–7 fold (e.g., histidine, isoleucine, phenylalanine). Interestingly, the abundance of asparagine during the sample homogenization decreased from 480 to 143 nmol/g, but during the incubation for 24 h, it increased to the level of 224 nmol/g. At the same time, the concentration of some amino acids remained relatively constant (glutamine, creatine) or even decreased (glutamate) during the incubation. The kinetic curves for several amino acids are shown in Figure 2, and the complete set of kinetic data is presented in Table S1.

*Organic acids*. The levels of many organic acids in the brain homogenate did not change during the incubation. We observed a significant (from threefold to tenfold) increase only for three compounds, acetate, γ-aminobutyrate, and pyroglutamate.

*Antioxidants.* We detected two compounds with well-known antioxidant properties, ascorbate, and GSH. The level of ascorbate remained almost unchanged during the first five hours of incubation, but after 24 h the concentration of ascorbate decreased by approximately 30%. The level of GSH decreased from 610 nmol/g at zero point to 80 nmol/g at 24 h.

*Alcohols, amines, sugars.* The concentration of glycerophosphocholine during the incubation decreased to almost zero level, while the levels of choline and phosphocholine increased. The increase was also observed for sugars glucose and mannose, and for alcohol glycerol.

*Nitrogenous bases, nucleotides, nucleosides.* Many compounds from this group underwent decomposition already during the sample homogenization, and at the zero time their level was either low (AMP, ADP, ATP) or below the detectable level (IMP, NAD, NADH, NADPH, GMP, UMP). During the incubation, the general trend in the evolution of compounds from this group is the decay of relatively complex molecules (ATP, ADP, AMP, adenosine, guanosine, inosine, uridine, coenzyme A) and the accumulation of products of their degradation (hypoxanthine, xanthine, uracil). The kinetic behavior for some compounds is non-monotonic. In particular, the levels of nucleosides (adenosine, guanosine, inosine) undergo strong growth during the sample homogenization and even at the initial stage of incubation (e.g., inosine, Figure 2), but then decay to almost zero level.

## 4. Discussion

In this work, we tried to evaluate the stability of metabolites in five biological media —serum, whole blood extract, whole blood homogenate, brain extract, and brain homogenate. We found that the degradation of compounds strongly depends on the media. Blood serum is a fluid most frequently used for metabolomic studies [27], and at 4 °C its metabolomic profile remains stable for 24 h. Such stability should be attributed to two main factors. Firstly, although serum contains a significant amount of proteins, most of them fulfill the transport function and do not cause metabolite transformations. Secondly, the abundance of unstable metabolites in serum (energy metabolites, antioxidants) is rather low, and in most cases, it is below the detectable NMR level. The addition of nucleotides to serum demonstrates that these compounds undergo degradation during the incubation at 4 °C, but at a very low rate.

The stability of metabolites in the blood and brain extracts is also very high. The concentrations of even the most unstable compounds such as ATP, ADP, CMP, GMP, NADH, and NAD do not change during the incubation. This finding indicates that the thermal reactions play a rather little role in the metabolite degradation and transformation, at least at relatively low temperatures (4 °C).

We compared two methods of whole blood extraction, based on cryogenic lysis (three cycles of freeze-thaw) and methanol lysis. The performance of both methods was similar. This indicates that the metabolic activity of blood enzymes is relatively low, and during the freeze-thaw cycles, the metabolomic changes do not have time to occur. The results of the whole blood homogenate incubation confirm this conclusion: changes in the metabolomic composition become apparent only after 1–2 h of incubation at 4 °C. The observed changes mostly correspond to energy metabolites directly involved in cellular energy generation and compounds participating in redox reactions: adenosine phosphates ATP, ADP, and AMP, NAD and NADH, GSH and GSSG, pyruvate, lactate, and so on. The observed changes should be attributed primarily to the enzymatic reactions catalyzed by intracellular blood proteins. However, one more possible pathway of metabolite degradation is oxidation by molecular oxygen. It has been shown that although the reaction between triplet oxygen and ground-state thiols (such as cysteine and glutathione) is spin forbidden, the presence of heavy metals in the solution may spin catalyze this reaction [35,36]. The enhanced level of iron in blood homogenates probably facilitates the reactions of GSH and NADH oxidation. From the data obtained, it is difficult to estimate the contribution of this channel to the total picture of metabolomic changes in blood homogenates.

The stability of blood metabolites has been a subject of several recent studies [21,22,37,38,39,40,41,42,43]. Haid et al. [39] reported that the metabolomic composition of human serum undergoes very slow changes during storage at even −80 °C. According to [22], the majority of serum metabolites are stable at both 4 °C and room temperature, although several compounds (allantoin, creatinine, glutamine) degrade much more rapidly than other metabolites. In paper [21], some metabolomic changes in the blood serum and plasma were detected after 3–6 h of incubation at both 0 °C and room temperature, but the incubation-induced changes in the metabolomic profiles were comparable with inter-individual variations. In work [40], changes in plasma metabolomic profiles were observed after the storage at room temperature for 15–30 min and at a cold temperature for 4–8 h. The most significant changes were detected in the levels of pyruvic acid and hypoxanthine. The author associated these effects with the metabolic activity related to the blood cells. According to [42], incubation of plasma at 25 °C causes faster and deeper sample degradation than that at 4 °C, with major changes corresponding to the levels of glucose, lactate, and pyruvate. In the paper of Loo et al. [43], it was shown that storage of plasma samples at 4 °C up to 48 h has minor effect on the sample metabolomic composition, while heating samples to 56 °C for 30 min induces significant metabolomic changes. The consensus between different authors is that though the blood metabolome is rather stable, some changes occur during the prolonged delay before the metabolism quenching, and the scale of this effect strongly depends on temperature. The results of our work related to the blood samples are in a good agreement with this conclusion.

The metabolite stability in the brain homogenate is much lower than that in the blood samples and in the brain extracts. The changes in the brain metabolomic composition proceed at different time scales. The most rapid changes observed in this work (at a minute time scale) correspond to energy metabolites attributed to the “Nitrogenous bases, nucleosides, nucleotides” group. These changes are well visible in the comparison of columns “Brain extract” and “Brain homogenate” at zero time in Table 2. The major process occurring within several minutes during brain homogenization is the decomposition of nucleotides (ATP, ADP, AMP, GTP, GMP, IMP, NAD, NADH, NAPH, and UMP) with the formation of intermediate and final products: cytidine, guanosine, inosine, hypoxanthine, uracil, uridine, xanthine, nicotinamide (Figure 2). Taking into account that the concentrations of all these compounds remain constant in the brain extracts, the observed effect should be attributed exclusively to the enzymatic reactions in the brain homogenate.

The strong difference between “Brain extract” and “Brain homogenate” at zero time does not mean that the measured metabolomic profiles of the brain extract at zero time precisely reflect the metabolomic state of in vivo brain. According to the literature data reviewed in works [23,44,45], significant post-mortem metabolomic changes occur already within several seconds after death due to ischemia caused by ceasing the blood supply to the brain. These changes primarily correspond to energy metabolites. Indeed, an inspection of the metabolomic data in Table 2 reveals elevated levels of lactate (6500 nmol/g) and AMP (1430 nmol/g), and very low concentrations of glucose (below detectable level), ATP (161 nmol/g), and ADP (236 nmol/g) in the brain extracts. In a normal in vivo brain, concentrations of these metabolites would be expected to be in the range of 1000–3000 nmol/g for glucose, lactate, and ATP [23,44,45,46,47,48,49]. Therefore, we can conclude that during tissue harvesting, practically all glucose is converted into lactate, and all ATP and ADP are converted into AMP. The importance of metabolism inactivation already at the stage of the brain harvest is well known in neurochemistry, but numerous examples given in a recent review by Dienel [23] indicate that in the metabolomics community, the use of inadequate methods of brain harvest is a common problem. To obtain the metabolomic profile most closely related to the living brain, one should use more sophisticated methods than decapitation followed by brain removal: microwave fixation [50], funnel freezing [51], or freeze blowing [52].

The influence of the sample preparation protocol on metabolome stability in the brain homogenates was studied in the paper [28]. The authors report that the ultrasonic homogenization of brain samples results in significant metabolomic changes. They also compared the NMR spectra of samples obtained by mechanical homogenization of the brain tissue in the methanol-chloroform mixture and in D_2_O followed by the sample centrifugation and lyophilization and did not observe noticeable differences between the spectra. The last observation contradicts our results. We should notice that the data on the most unstable compounds—nucleotide triphosphates, NAD, NADH—are absent in the work [28], which probably explains this contradiction.

**Figure 3:**
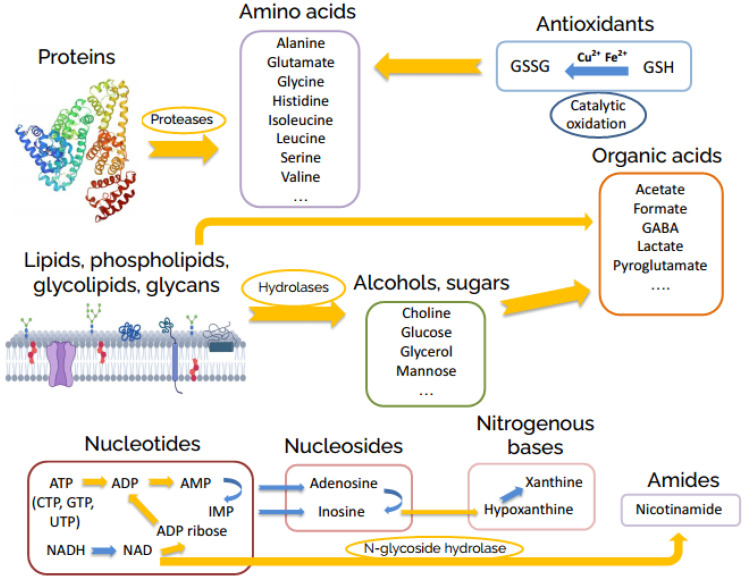
General scheme of post-mortem metabolite transformations in rat brain homogenates.

The metabolomic changes in the brain homogenate continue at the hour time scale. These changes are also induced by enzymatic reactions and include: almost complete decomposition of nucleotides; the decay of intermediate products of the nucleotide decomposition formed at the initial stage—ADP ribose, adenosine, guanosine, inosine, uridine; strong increase of the level of the majority of amino acids due to protease-catalyzed protein hydrolysis; glycoside hydrolase-induced formation of glucose, mannose, choline, glycerol; and the accumulation of final reaction products such as acetate, γ-aminobutyrate (GABA), and pyroglutamate. We depicted the supposed general scheme of reactions occurring in the brain homogenates in Figure 3.

Due to its clinical importance, the stability of metabolites in cerebrospinal fluid (CSF) is studied much better [53,54,55]. It was found that the CSF metabolome remains stable at room temperature for two hours if the cellular elements are removed by centrifugation immediately after the sample collection [55]. Otherwise, changes in the concentrations of several amino acids and organic acids during the incubation at room temperature occur [53]. In the recent report [56] it was shown that the CSF metabolome remains rather stable at 4 °C, but undergoes significant changes at room temperature. This indicates that in the sense of metabolic activity and metabolome stability, CSF and blood are similar. The data on the stability of the CSF metabolome are in a fair agreement with our results obtained for the blood samples: the metabolomic profiles of the blood plasma and whole blood extract do not change during the incubation at 4 °C for 24 h, while metabolites in the whole blood homogenate undergo slow degradation.

## 5. Conclusions

Concluding, the results obtained in this work demonstrate that if the sample preparation is performed at relatively low temperatures (4 °C or below), the enzymatic reactions play a much more important role in the metabolomic changes than the spontaneous thermal reactions. The extracts from biological tissues do not contain enzymes, and at 4 °C their metabolomic compositions remain constant for at least 24 h. Human serum also contains a rather low amount of metabolically active proteins and 24 h of incubation at 4 °C results in only minor changes in the serum metabolome. The blood and brain homogenates contain intracellular enzymes released during cell lysis, and without metabolism quenching, these enzymes cause significant variations in the homogenate metabolomic composition. In the whole blood homogenates, these changes are relatively slow, and they become apparent only after several hours of incubation at 4 °C. For that reason, both methods used in the blood homogenization, cryogenic lysis and methanol lysis, gave similar results. The brain tissue is much more metabolically active than human blood, and significant metabolomic changes occur almost immediately after death. This indicates that the method of brain tissue harvest is critically important for correct metabolomic profiling, especially concerning energy metabolites. Our measurements demonstrate that decapitation followed by brain removal and snap freezing induces too large a lag between the animal death and brain freezing to preserve labile metabolites such as ATP, ADP, AMP, glucose, and lactate. Our results also show that in pre-analytical sample preparation, the metabolite transformation occurs within several minutes during the sample homogenization without metabolism quenching in advance. Therefore, in the metabolomic analysis of such tissues as the brain, reliable data can be obtained only if the correct method of brain harvest is used, and metabolism is quenched at the earliest stage of the sample preparation—either immediately after the sample collection, or during the thawing of preliminary snap-frozen brain samples.

It is important to notice that we performed the quantitative metabolomic analysis with the use of the NMR method and determined the concentrations of only 50–70 most abundant compounds. The LC-MS method is more sensitive; it allows for the detection and quantification of hundreds of metabolites. However, since the analysis present in this work includes all important classes of metabolites, unstable ones, in particular, we believe that the results of this work are applicable to both NMR and LC-MS-based metabolomic studies.

## Figures and Tables

**Table 1:** Concentrations of metabolites (in units of µM) in serum, whole blood extract, and whole blood homogenate at zero time and after 24 h of incubation at 4 °C. Abbreviations: Ac-Carnitine—acetylcarnitine; ADP—adenosine diphosphate; AMP—adenosine monophosphate; ATP—adenosine triphosphate; GSH—glutathione reduced; GSSG—glutathione oxidized; Gl-PhCholine—glycerophosphocholine; IMP—inosine monophosphate; LOD—level of detection; NADP—nicotinamide adenine dinucleotide phosphate; PhCholine—phosphocholine.

Sample Type	Serum	Extract		Homogenate	
Time, h	0	0	24	0	24
**Amino acids and their derivatives, peptides**					
Alanine	471 ± 22	284 ± 19	275 ± 15	330 ± 60	400 ± 50
Asparagine	120 ± 50	105 ± 9	94 ± 5	105 ± 9	94 ± 6
Aspartate	260 ± 40	109 ± 17	90 ± 7	95 ± 15	102 ± 12
Ac-Carnitine	10 ± 3	19 ± 1	16.5 ± 0.4	14.9 ± 2.5	15.3 ± 2.2
Betaine	45.4 ± 2.9	51.9 ± 2.8	61.2 ± 2.9	50.9 ± 1.1	55 ± 6
Creatine	43 ± 8	160 ± 7	172 ± 12	138 ± 16	144 ± 28
Creatinine	97 ± 11	90 ± 4	87 ± 6	86 ± 5	92 ± 9
Ergothioneine	<LOD ^1^	154 ± 25	98 ± 4	70 ± 50	70 ± 60
Glutamate	160 ± 60	183 ± 11	213 ± 12	230 ± 23	259 ± 16
Glutamine	680 ± 70	520 ± 30	530 ± 40	500 ± 80	460 ± 90
Glycine	210 ± 4	241 ± 21	233 ± 13	250 ± 40	353 ± 29 *
GSH	<LOD	383 ± 19	175 ± 29 *	430 ± 50	46 ± 11 *
GSSG	671 ± 9	575 ± 17	420 ± 50 *	554 ± 25	810 ± 50 *
Histidine	114 ± 28	73.3 ± 1.2	77 ± 4	75 ± 8	78.4 ± 2.1
Isoleucine	65 ± 3	61 ± 7	51 ± 5	50 ± 9	63.3 ± 1.9 *
Leucine	122 ± 7	107 ± 7	105 ± 6	104 ± 9	118.8 ± 2.8 *
Lysine	189 ± 25	134 ± 8	131 ± 12	135 ± 7	142 ± 18
Ornithine	91 ± 6	87 ± 8	78 ± 4	126 ± 17	153 ± 6
Phenylalanine	61 ± 17	49.6 ± 1.9	49 ± 3	50.4 ± 0.8	62 ± 4 *
Proline	356 ± 17	192 ± 17	161 ± 5	214 ± 27	221 ± 23
Serine	110 ± 50	75 ± 11	62 ± 6	76 ± 10	117 ± 7 *
Threonine	178 ± 7	136 ± 14	135 ± 8	153 ± 7	165 ± 11
Tryptophan	70 ± 9	38.2 ± 2.6	38.5 ± 0.9	40 ± 5	50 ± 5
Tyrosine	66 ± 14	48.1 ± 2.1	48 ± 3	54 ± 3	62 ± 4
Valine	186.0 ± 1.2	186 ± 17	182 ± 13	151 ± 8	186.3 ± 2.9 *
**Organic acids**					
Acetate	580 ± 40	66 ± 11	104 ± 6 *	67 ± 20	92 ± 9 *
α-Aminobutyrate	26 ± 11	13.7 ± 1	15.97 ± 0.15	12.4 ± 1.4	15.1 ± 1.3
β-Hydroxybutyrate	33 ± 11	27.2 ± 2.6	22.4 ± 1.1	17 ± 6	16.3 ± 1.5
Formate	69 ± 29	46 ± 20	67 ± 4	51 ± 6	44 ± 11
Fumarate	4.3 ± 2.0	1.6 ± 0.3	1.8 ± 0.3	2.4 ± 0.4	4 ± 1
Isobutyrate	6.6 ± 2.5	7.4 ± 1.2	9.1 ± 0.9	6.1 ± 1.9	8.8 ± 1.1
α-Ketoisovalerate	13 ± 5	9.2 ± 1.2	7.6 ± 1.2	6 ± 0.21	6.6 ± 1.6
Lactate	2770 ± 60	1230 ± 30	1270 ± 90	1210 ± 50	2120 ± 70 *
Pyroglutamate	110 ± 70	13.1 ± 2.4	22 ± 4	20 ± 4	77 ± 6 *
Pyruvate	11 ± 6	17 ± 4	29.8 ± 1.8 *	12 ± 7	86 ± 15 *
Succinate	3.2 ± 2.7	3.91 ± 0.13	4.6 ± 0.6	3.9 ± 0.3	9.4 ± 1.6
**Alcohols, amines, amides, sugars**					
Acetone	11.4 ± 1.2	4.9 ± 0.5	4.3 ± 0.5	3.7 ± 1.2	3.7 ± 1.5
Choline	13.8 ± 2.9	10.2 ± 2.0	11.0 ± 2.0	16.7 ± 2.7	33.7 ± 2.8 *
Dimethylamine	18.3 ± 1.3	8.1 ± 0.4	7.3 ± 0.4	7.4 ± 0.4	7.7 ± 0.9
Glucose	3740 ± 60	3700 ± 400	4400 ± 900	3390 ± 110	5190 ± 70 *
Gl-PhCholine	22.1 ± 1.0	21.8 ± 0.6	19.3 ± 1.7	18.3 ± 0.5	17.1 ± 2.0
Mannose	33 ± 15	42.0 ± 2.0	31.2 ± 2.3 *	41 ± 3	<LOD *
Nicotinamide	<LOD	3.5 ± 2.5	5.1 ± 0.9	39 ± 4 ^3^	130 ± 4 *
PhCholine	4.08 ± 0.23	5.7 ± 0.7	5.2 ± 1	7.1 ± 1.3	8.7 ± 1.1
**Nitrogenous bases, nucleotides, nucleosides**					
ADP + ATP	<LOD	664 ± 14	460 ± 50 *	560 ± 110	200 ± 50 *
ADP ribose	<LOD	7 ± 6	11 ± 4	41 ± 3 ^3^	78 ± 5 *
AMP	<LOD	8.9 ± 0.7	15.6 ± 1.3 *	33 ± 5	220 ± 140 *
Hypoxanthine	<LOD	2.1 ± 2.1	3.2 ± 0.6	3.9 ± 0.8	21 ± 7 *
IMP	<LOD	3.2 ± 1.9	2.8 ± 1.6	6.4 ± 1.5	133 ± 7 *
NAD	<LOD	22 ± 6	28.1 ± 0.3	90 ± 4 ^3^	<LOD *
NADH	<LOD	4.6 ± 2.0	0.8 ± 1.0 *	22.8 ± 2.2 ^2^	1.3 ± 2.8 *
NADP	<LOD	14.9 ± 1.8	12.8 ± 1.1	6.5 ± 1.6	8 ± 0.4

^1^ LOD for a singlet signal from a single proton can be estimated as 2 µM; ^2^ 100 µM NADH were added to homogenates before incubation; ^3^ NAD, ADP ribose, and nicotinamide in homogenates at zero time are partly formed due to decomposition of manually added NADH. * Metabolite concentration changed significantly (*p* < 0.05) after 24 h of incubation.

**Table 2:** Concentrations of metabolites (in units of nmol/g) in extracts and homogenates of the rat brain at zero time and after 24 h of incubation at 4 °C.

Sample type	Extract		Homogenate	
Time, h	0	24	0	24
**Amino acids and their derivatives, peptides**				
Alanine	384 ± 15	388 ± 10	530 ± 40	1043 ± 29 *
Asparagine	480 ± 100	360 ± 150	143 ± 11	224 ± 5 *
Aspartate	2270 ± 80	2260 ± 70	3200 ± 150	8030 ± 240 *
Ac-Carnitine	25 ± 5	23.7 ± 2.4	22 ± 6	8.4 ± 1.3 *
Carnosine	74.4 ± 2.2	69.9 ± 1.8	85 ± 8	89 ± 6
Creatine	7490 ± 190	7540 ± 250	8570 ± 130	7600 ± 280 *
Glutamate	7430 ± 160	7670 ± 230	8000 ± 400	3170 ± 150 *
Glutamine	4110 ± 80	4150 ± 150	5020 ± 110	4700 ± 150
Glycine	830 ± 120	820 ± 110	940 ± 50	2550 ± 190 *
GSH	740 ± 70	690 ± 80	610 ± 50	80 ± 30 *
GSSG	170 ± 30	168 ± 25	<LOD	<LOD
Histidine	43.9 ± 2.5	49 ± 5	69 ± 8	370 ± 40 *
Isoleucine	17.5 ± 2.4	20.9 ± 2.7	33 ± 4	185 ± 15 *
Leucine	65 ± 7	68 ± 5	113 ± 14	510 ± 60 *
N-acetylaspartate	5630 ± 90	5650 ± 100	6200 ± 270	1210 ± 50 *
Pantothenate	15.3 ± 0.8	17.1 ± 0.9	23.7 ± 2.8	42 ± 5 *
Phenylalanine	81.9 ± 1.6	80 ± 12	94 ± 11	700 ± 60 *
Serine	510 ± 40	493 ± 16	550 ± 30	1248 ± 16 *
Threonine	420 ± 70	430 ± 50	340 ± 50	610 ± 80 *
Tryptophan	16 ± 5	20 ± 4	27.5 ± 2.7	68 ± 7 *
Tyrosine	54 ± 4	51.2 ± 1.9	76 ± 4	231 ± 17 *
Valine	42 ± 5	44 ± 4	58 ± 8	197 ± 8 *
**Organic acids**				
Acetate	158.7 ± 1.4	168 ± 10	390 ± 90	4050 ± 100 *
α-Aminobutyrate	5.4 ± 1.6	8.6 ± 2.3	5.6 ± 1.0	5.7 ± 1.2
γ-Aminobutyrate	1710 ± 180	1670 ± 150	2800 ± 400	7890 ± 230 *
Ascorbate	640 ± 40	644 ± 28	844 ± 10	517 ± 28 *
Formate	130 ± 11	132 ± 9	135 ± 6	132 ± 15
Fumarate	12.7 ± 2.1	15.4 ± 0.8	76.1 ± 2.5	48.8 ± 0.7 *
Isobutyrate	8.4 ± 1.6	6.6 ± 1.1	5.2 ± 1.3	5.6 ± 1.9
Lactate	6500 ± 300	6500 ± 300	8000 ± 210	7040 ± 280 *
Pyroglutamate	<LOD	<LOD	96 ± 21	473 ± 11 *
Pyruvate	5.3 ± 1.6	11.3 ± 1.3 *	5.5 ± 2.3	5.7 ± 1.3
Succinate	416 ± 12	416 ± 8	16.8 ± 2.0	33.6 ± 0.6 *
Taurine	3060 ± 140	3060 ± 130	3800 ± 50	3622 ± 27 *
**Alcohols, amines, amides, sugars**				
Choline	52 ± 6	52 ± 3	250 ± 80	691 ± 14 *
Glucose	<LOD	<LOD	470 ± 60	1210 ± 40 *
Gl-PhCholine	650 ± 40	650 ± 40	610 ± 60	10 ± 8 *
Glycerol	164 ± 15	158 ± 9	320 ± 40	1060 ± 40 *
myo-Inositol	6190 ± 160	6190 ± 160	6763 ± 23	6763 ± 23
scyllo-Inositol	96 ± 6	90 ± 14	84.8 ± 2.6	86 ± 4
Mannose	<LOD	<LOD	58 ± 13	229.3 ± 1.1 *
Nicotinamide	80 ± 30	81 ± 21	159 ± 22	110 ± 24
PhCholine	305 ± 16	301 ± 19	480 ± 60	833 ± 14 *
Phosphoethanolamine	800 ± 30	830 ± 40	800 ± 17	600 ± 30 *
**Nitrogenous bases, nucleotides, nucleosides**				
Adenosine	193 ± 6	201 ± 16	500 ± 400	7 ± 6 *
ADP	236 ± 17	230 ± 40	53 ± 11	10.9 ± 1.9 *
ADP ribose	110 ± 30	120 ± 30	120 ± 90	4 ± 3 *
AMP	1430 ± 70	1430 ± 60	81 ± 27	32 ± 6 *
ATP	161 ± 28	144 ± 15	60 ± 50	12 ± 3 *
Coenzyme A	26.3 ± 0.6	25.3 ± 2.8	32.9 ± 1.1	2.4 ± 0.8 *
Cytidine	19 ± 8	24 ± 6	46 ± 6	67 ± 6 *
GMP	196 ± 27	191 ± 18	<LOD	<LOD
GTP	136 ± 7	127 ± 2.9	69 ± 16	21 ± 5 *
Guanosine	8.5 ± 1.2	5 ± 3	35 ± 23	1 ± 0.6 *
Hypoxanthine	<LOD	<LOD	290 ± 120	1230 ± 70 *
IMP	140 ± 40	140 ± 30	<LOD	<LOD
Inosine	120 ± 16	110 ± 18	1100 ± 400	100 ± 9 *
NAD	160 ± 40	167 ± 30	<LOD	<LOD
NADH	19.5 ± 2.8	15 ± 3	<LOD	<LOD
NADPH	10.2 ± 1.1	4.3 ± 1.3 *	<LOD	<LOD
UMP	90 ± 6	95 ± 9	<LOD	<LOD
Uracyl	<LOD	<LOD	44 ± 12	261 ± 6 *
Uridine	48 ± 4	49 ± 3	128 ± 20	12 ± 10 *
Xanthine	<LOD	<LOD	117 ± 30	280 ± 13 *

* Metabolite concentration changed significantly (*p* < 0.05) after 24 h of incubation.

## Data Availability

Raw NMR spectra, description of specimens and samples, metabolite concentrations and the preliminary metabolomic analysis are available at our Animal Metabolite Database repository, Experiment ID 230 and 231 (https://amdb.online/amdb/experiments/list/, accessed on 1 July 2022). All other data are available from the corresponding author upon request.

## Supplementary Materials

The following supporting information can be downloaded at: https://www.mdpi.com/article/10.3390/metabo12090811/s1, Figure S1: NMR spectrum of protein-free lipid-free extract from the whole human blood; Figure S2: NMR spectrum of protein-free lipid-free extract from the rat brain; Table S1: Kinetic data on metabolite concentrations in the human blood homogenates and rat brain extracts and homogenates during the incubation at 4 °C.
