# Supplementary material for: Stability of Metabolomic Content during Sample Preparation: Blood and Brain Tissues
Source: Metabolites. 2022 Aug 29;12(9):811. doi: 10.3390/metabo12090811 (PMC9505456; doi:10.3390/metabo12090811)
Supplement: Supplementary file 1 [file metabolites-12-00811-s001.zip › SI_blood-brain.pdf]

Stability of metabolomic content during sample preparation: blood and brain tissues

Maxim V. Fomenko [1,2], Lyudmila V. Yanshole [1], Yuri P. Tsentalovich [1,*]

[1] Laboratory of Proteomics and Metabolomics, International Tomography Center SB RAS, Institutskaya 3a, Novosibirsk 630090, Russia
[2] Novosibirsk State University, Pirogova 2, Novosibirsk 630090, Russia

* e-mail: yura@tomo.nsc.ru

Content:

Figure S1. NMR spectrum of protein-free lipid-free extract from the whole human blood. The signals used for metabolite quantification are marked by bold.
Figure S2. NMR spectrum of protein-free lipid-free extract from the rat brain. The signals used for metabolite quantification are marked by bold.

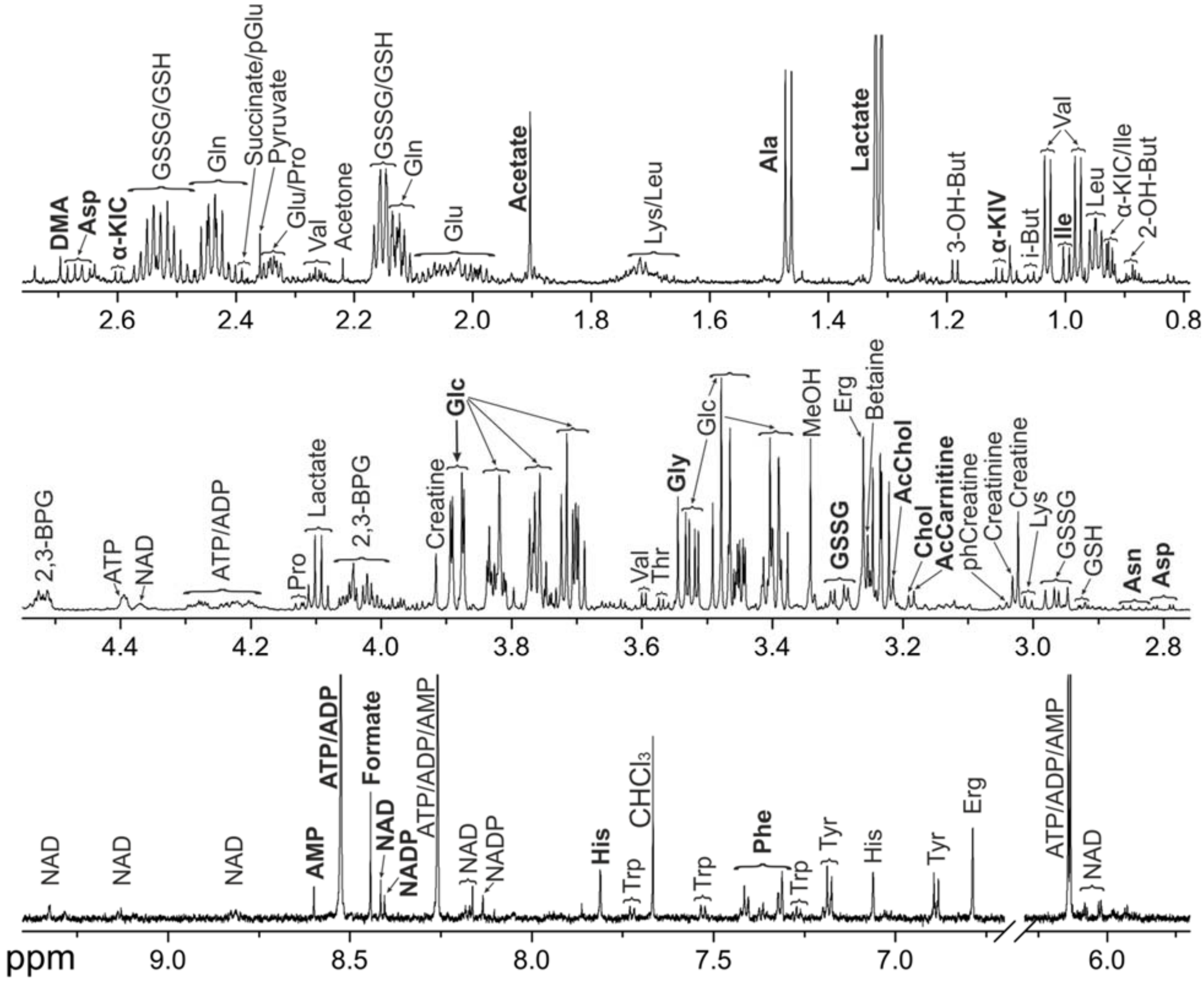

Figure S1. NMR spectrum of protein-free lipid-free extract from the whole human blood. The signals used for metabolite quantification are marked by bold.

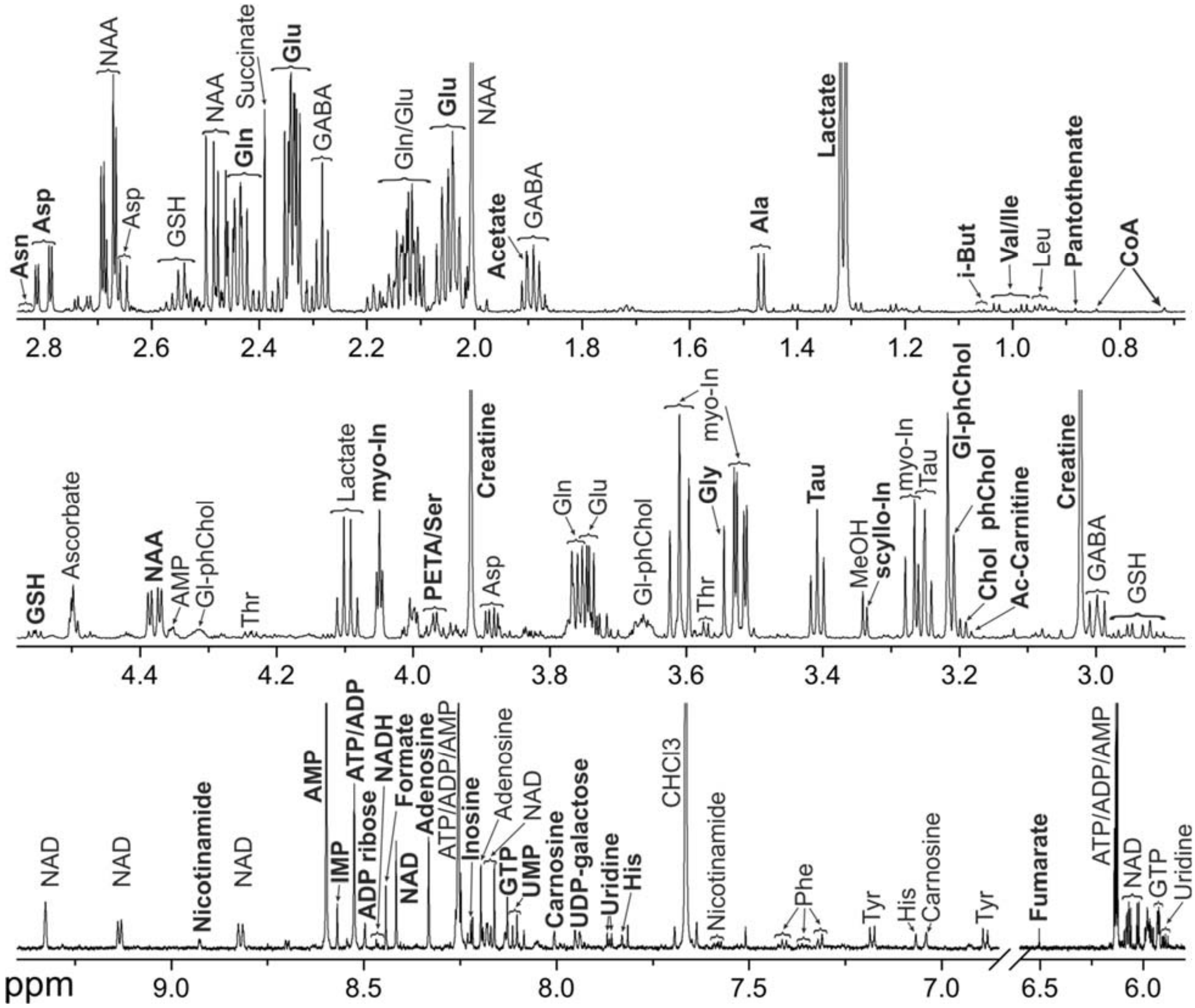

Figure S2. NMR spectrum of protein-free lipid-free extract from the rat brain. The signals used for metabolite quantification are marked by bold.
